# Novel Strategies to Improve the Anticancer Action of 5-Fluorouracil by Using Drug Delivery Systems

**DOI:** 10.3390/molecules13102340

**Published:** 2008-10-01

**Authors:** José L. Arias

**Affiliations:** Department of Pharmacy and Pharmaceutical Technology, Faculty of Pharmacy, University of Granada, Campus Universitario de Cartuja s/n, 18071 Granada, Spain; E-mail: jlarias@ugr.es; Tel.: (+34) 958 24 39 00; Fax: (+34) 958 24 89 58

**Keywords:** Biodegradable polymers, Cancer treatment, Drug carriers, 5-Fluorouracil, Vesicular systems

## Abstract

Because of the fundamental importance of new therapeutic routes for cancer treatment, a number of systems based on colloidal particles as vehicles for the delivery of the anticancer drug 5-fluorouracil have been devised. The target is always to provide the proper dose of the antitumor agent only at the desired locus of action, thus reducing the unwanted side effects. In this review, the main strategies and the more significant results in the development of 5-fluorouracil carriers for cancer treatment are discussed.

## 1. Introduction

Apart from surgery, radiation and biologic therapies (immunotherapy and hormonal therapy), the therapeutic strategies presently used in the clinical practice for cancer treatment are focused on cytotoxic drugs as the main form of chemotherapy for cancer. Cytotoxic drugs are very heterogeneous chemical compounds that treat cancer primarily by being toxic to cells that are rapidly growing and dividing. Because cancer cells often undergo rapid growth and proliferation, they are preferentially killed by these agents. These substances are conventionally administered intravenously in the form of free drug solutions. Despite the long history of their use and the development of numerous new multiple drug regimens to improve clinical success, treatment failure is still frequently encountered and even in patients with malignancies that are more sensitive to chemotherapeutic agents (e.g., breast cancer), the clinical outcomes are usually below expectation.

In comparison to other drug classes, cytotoxic anticancer drugs present unique problems that come primarily from the relative lack of specificity of their systemic biodistribution and the subsequent side effects provoked by the drug attacking both healthy and target cells. In addition, their unfavorable pharmacokinetics implies the administration of high drug doses, and imposes on patients a rigorous schedule for reaching the desired therapeutic effect. These unwanted properties exhibited by free drugs can be summarized in the following points: *i*) the hydrophobic character of many drugs promotes their precipitation in aqueous media; *ii*) they suffer of poor selectivity for target tissues; *iii*) they undergo biodistribution in a large body volume with severe side effects in sensitive nontarget tissues; *iv*) their inadvertent extravasation can produce damage in healthy tissues; *v*) molecular drugs suffer rapid clearance; *vi*) their *in vivo* degradation implies the necessity of high dose administration; and, finally, *vii*) their susceptibility to induce drug resistance [1, 2].

5-Fluorouracil (5-FU or 5-fluoro-2,4-pyrimidinedione) is an antimetabolite of the pyrimidine analogue type, with a broad spectrum of activity against solid tumors (of the gastrointestinal tract, pancreas, ovary, liver, brain, breast, etc.), alone or in combination chemotherapy regimes. Due to its structure, 5-FU interferes with nucleoside metabolism and can be incorporated into RNA and DNA, leading to cytotoxicity and cell death. One of the main reasons that had greatly limited the clinical applications of 5-FU over the past 50 years is the development of drug resistance by tumor cells. For example, the overall response rate for advanced colorectal cancer of 5-FU alone is still only ≈ 10 %, and the combination of 5-FU with other antitumor drugs has merely improved the response rates to just ≈ 45 %. In addition, because of its high rate of metabolism in the body, the maintenance of a therapeutic serum concentration requires the continuous administration of high doses, that if induce concentrations above a certain limit produces a severe toxic effect [3, 4].

The therapy of 5-FU can be improved and its toxicity overcome (including myelosupression and gastrointestinal, haematological, neural and dermatological adverse side effects) diminished by facilitating the specific accumulation of this anticancer agent in the tumor infected regions with prolonged exposure of the cells to this agent. In this sense, the association of anticancer drugs to delivery systems has been an interesting approach to selectively delivering these active agents and, at the same time, reducing their toxicity. Another benefit of 5-FU localization in the targeted tissue could be an improvement in its pharmacokinetic profile (short biological half life, and non-uniform oral absorption as a consequence of its metabolism by the enzyme dihydropyrimidine dehydrogenase or uracil reductase). Additionally, and due to the protection of this active agent by the carrier, another advantage might be the reduction in the formation of cardiotoxic 5-FU degradation compounds in the basic medium of the injected vials [4].

For these purposes, a suitable 5-FU delivery system should have the following properties: *i*) physical stability; *ii*) small size (at least less than 1 μm, if they are not intended to be used as implants), to allow capillary distribution and uniform perfusion at the desired target site; *iii*) the ability to carry adequate amounts of this drug, without excessive loading of the organism with foreign material; *iv*) protection from degradation; *v*) absence of low storage and drug leakage problems; *vi*) controllable (or predictable) 5-FU release rates from the carrier at the desired target site; *vii*) surface properties permitting maximum biocompatibility and minimal antigenicity; and *viii*) biodegradability with either elimination or minimized toxicity of breakdown products [5]. Described below are the main 5-FU colloidal carriers and strategies developed for its delivery to cancer. The use of polymer conjugates or copolymers in the design of 5-FU delivery systems is also considered, keeping the related information along with that of their corresponding polymeric constituents.

## 2. 5-Fluorouracil delivery systems

### 2.1 Biodegradable polymeric particles

#### 2.1.1 Alginate beads

Polysacharides such as alginate have been extensively used in the food, cosmetics, pharmaceutical and biomedical industries due to their gel forming properties in the presence of multivalent cations. Alginate, a naturally occurring copolymer of glucuronic acid and manuronic acid, is widely used for pharmaceutical applications. Specifically, the simple aqueous-based gel formation of sodium alginate in the presence of divalent cations such as Ca^2+^ has been used for drug delivery.

Spherical alginate beads of around 1 – 2 mm containing 5-FU had been prepared by the gelation of alginate with calcium cations. Briefly, these beads can be prepared by extruding a 1 – 2 % (w/v) sodium alginate aqueous solution and 5-FU through a 22-gauge needle into 100 mM CaCl_2_ aqueous solution [6]. The encapsulation efficiencies obtained for this hydrophilic drug reached to 10 % and the authors found that as the drug-load was increased, larger beads were obtained, in which the resultant beads contained higher 5-FU content. Drug release showed a biphasic profile and is finished after 7 hours. Finally, it was observed that the amount of 5-FU released from alginate beads increased with decreasing alginate concentrations.

#### 2.1.2 Poly(ε-caprolactone)

Poly(ε-caprolactone) is a biodegradable polyester widely employed in the drug delivery field. It is a non-toxic, highly hydrophobic crystalline polymer with slow degradation, both *in vitro* and *in vivo*. Several 5-FU delivery systems based on this polymer had been developed for cancer treatment.

Martini *et al*. [7] synthesized a novel triblock copolymer of ε-caprolactone and ethylene oxide by employing the “hot-melt” method of microencapsulation. Non-linear 5-FU release kinetics were observed in the 75 – 250 μm particles, with a pronounced “burst release” associated with the presence of the drug at the surface of the microspheres.

This type of particles had also been used in the design of implantable matrices loaded with 5-FU. With that aim, it was proposed the preparation of the biodegradable poly(ε-caprolactone)-block-poly(ethylene glycol)-block-poly(ε-caprolactone) (PCL-PEG-PCL) by reacting PEG of 35 kDa with ε-caprolactone at 185 °C in bulk without catalyst and under vacuum [8]. The copolymer formation occurs through the well-known ring-opening mechanism, where an active hydrogen atom of the preformed PEG causes a selective acyl-oxygen cleavage of an ester group of the monomer ring. After the initial formation of an intermediate bishydroxy-diester, a step-by-step addition of monomer units occurs with formation of two external polyester blocks, the length of which depends on the amount of cyclic ester monomer used in the feed. The results obtained by these authors lead to a possibility of using such copolymer as a “time-delayed” 5-FU releasing system: a fast release of the anticancer drug in the first 10 hours is followed by a slow release phase, and finally reaches a limiting value after 24 hours.

This copolymer had also been used in the preparation of 5-FU loaded swellable matrices. The procedure involves dry compression of mixtures of the drug and copolymer using low compressional forces. The analysis of the release data revealed a predominantly diffusion controlled mechanism. Non-zero order release kinetic was observed, consequence of a gradual increase in the gel layer thickness during release [9].

Poly(D,L-lactide-ran-ε-caprolactone)-poly(ethylene glycol)-poly(D,L-lactide-ran-ε-caprolactone) triblock copolymers are also proposed for 5-FU delivery. This copolymer can be prepared by ringopening polymerization of D,L-lactide and ε-caprolactone in the presence of PEG, using Zn L-lactate as initiator. High 5-FU loading efficiencies are obtained following this method (> 90 %). The thermal behavior of the particles, such as melting temperature and melt viscosity, shows their potentiality as injectable drug-delivery device. As the melting temperature approaches room temperature, a less porous inner structure is formed compared to the one with higher melting temperature, resulting in slower release rate. The variation of composition in the triblock copolymer, such as PEG and D,L-lactyl/ε-caproyl ratio, lead to diverse drug release behaviors as well as different physicochemical characteristics (thermal behavior) and morphological changes. It is also observed that as the PEG content is increased, the drug release rate is increased due to the more and easier matrix swelling . Therefore, the control of all of these variables allows obtaining a biphasic release profile suitable for cancer treatment [10].

#### 2.1.3. Chitosan

Chitosan, obtained by deacetylation of chitin, is a widely accepted hydrophilic polymer due to its biocompatibility, biodegradability and mucoadhesion properties. Due to its insolubility at physiological pHs, there is a problem of precipitation leading to limited use in the case of applications related to the intravenous route. However, keeping in view the advantages associated with chitosan, the chitosan derivatives have been synthesized for biological applications, of which the glycol chitosan has received considerable attention due to its wide solubility range. The preparation procedure of the 5-FU-loaded chitosan particles is a key-role factor that must be carefully selected in order to achieve good encapsulation results and release profiles. For example, the formulation of chitosan as concentric multi-walled microspheres does not induce high 5-FU loading ratios (≈ 3 %). In addition, the release of the drug from the particles (270 – 370 μm) is very fast (≈ 80 % after 80 min) [11].

Dubey and Parikh [12] had synthesized chitosan microspheres loaded with 5-FU by using a procedure based on a chemical denaturation method that allows to prepare microparticles with a mean diameter of around 12 μm. The maximum 5-FU loading achieved is ≈ 50 %, and the drug release followed the Higuchi´s square-root model. Finally, as it is often the case, it was observed a positive effect of the concentration of the water-soluble drug on the loading.

**Figure 1:**
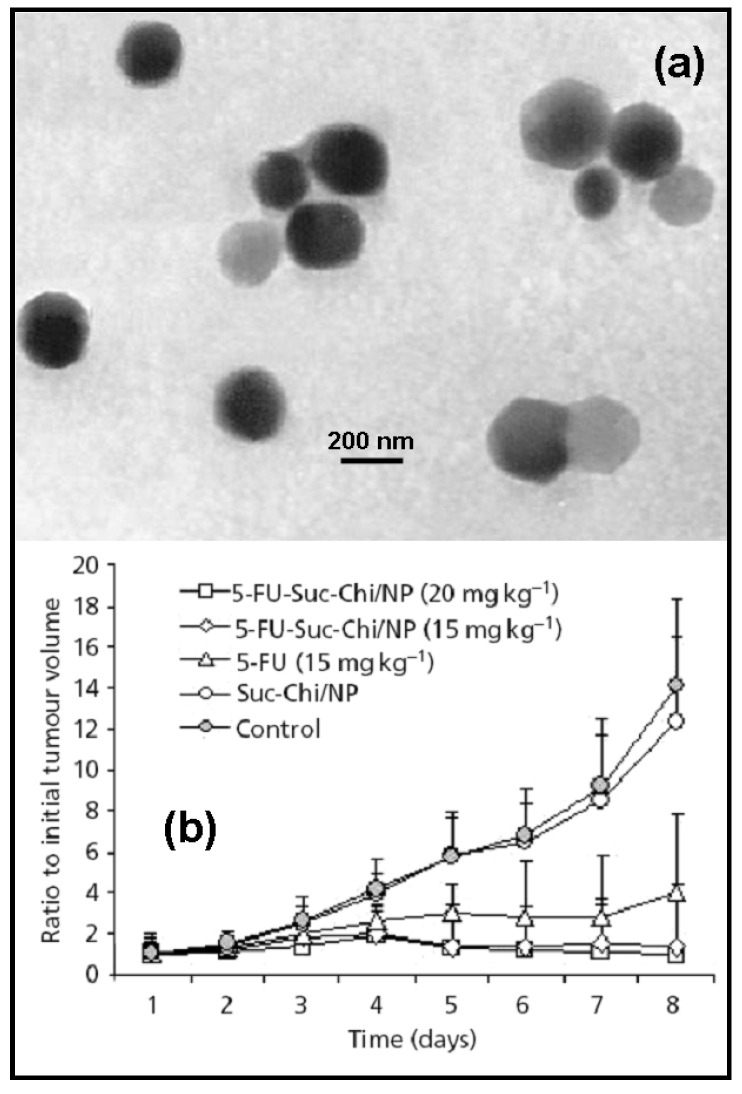
TEM photographs of blank Suc-Chi NPs (a), and tumor-growth-inhibitory effects of 5-FU-loaded *N*-succinylchitosan nanoparticles against Sarcoma 180 solid tumor (b). Data represent mean ± S.D., n = 6. Reprinted with permission from Ref. [14]. Copyright Pharmaceutical Press (2006).

Chitosan particles had been successfully assayed *in vivo* with very promising tumor inhibition rates. For example, 5-FU-loaded chitosan-polyaspartic acid nanoparticles of ≈ 200 nm can be synthesized by ionic gelation of chitosan, obtaining a loading efficiency of ≈ 40 %. These nanoparticles developed a sustained release during 6 days and the observed tumor inhibition rate was much higher than the anticancer drug alone [13]. Yan *et al*. [14] also observed good antitumor activity against Sarcoma 180 solid tumor and mild toxicity in 5-FU-loaded *N*-succinylchitosan nanoparticles prepared by an emulsification solvent diffusion method (average diameter ≈ 250 nm and, loading capacity and highest extent of release: 19 %, and 61 % at 24 h., respectively) (Figure 1).

New nonstoichiometric polyelectrolyte complex nanoparticles based on the complexation of chitosan (CS) and polyaspartic acid sodium salt (PAsp) had been proposed for 5-FU delivery. Nanoparticles made of polyelectrolytes complexation are formed by interaction between macromolecules that carry oppositely charged ionisable groups. In few words, 5-FU loaded nanoparticles were prepared by dropping a mixture solution of 5-FU and CS into a PAsp solution, and nanoparticles of 150 – 220 nm were obtained with an encapsulation efficiency of ≈ 45 %. *In vitro* and *in vivo* experiments indicated that the drug-loaded CS-PAsp nanoparticles presented a sustained release of 5-FU compared to the 5-FU solution and that the areas under curve (AUC) were increased by about four times [15].

Chitosan and its derivatives can also be used in the preparation of 5-FU-loaded hydrogels. For example, hydrogel microspheres of chitosan and pluronic F-127 (110 – 380 μm) are prepared by an emulsion-crosslinking method, employing glutaraldehyde as a crosslinker. The encapsulation of the drug achieved an efficiency of 86 % and the *in vitro* release studies indicated the dependence of the release rate on the extent of crosslinking, the drug loading and the amount of pluronic F-127 used. The slow release observed was extended up to 24 hours [16].

Water-soluble derivatives of chitosan, such as N-(2-carboxybenzyl)chitosan, had been proposed as potential pH-sensitive hydrogels for colon-specific 5-FU delivery. The studies on the release of 5-FU showed an initial burst release of ≈ 25 % at both pHs (7.4 and 1.0) within the first 15 min. About 20 min. later, the hydrogels serve as diffusion barriers and the drug is released mainly by a diffusion mechanism. As expected, the amount of 5-FU released under acidic conditions (pH 1.0) was relatively low. Only about 40 % of the loaded drug was released after 12 h, due to the comparatively low swelling ratio of the hydrogel at pH 1.0. At pH 7.4, the release of 5-FU increased significantly along with the swelling of the hydrogel network. Approximately 90 % of the loaded drug was released after 10 hours in pH 7.4 buffer [17].

Chitosan had also been used to increase the controlled release behavior of other drug delivery systems. In these cases, chitosan is used for preparation of polymeric coatings that slow down the loose of the drug. As an example, 5-FU-loaded polylactic acid microspheres coated with chitosan had been prepared as biodegradable carriers for cerebral tumors. Briefly, an emulsion of 5-FU (in water) and polylactic acid dissolved in an acetone-dichloromethane mixture is poured into an aqueous solution of chitosan for the spontaneous formation of microspheres (10 – 25 μm). The maximum drug loading value achieved is ≈ 65 % and the drug release behavior exhibited a biphasic pattern: an initial burst release (≈ 25 %), followed by a constant slow release profile during 30 days [18].

#### 2.1.4. Eudragit^®^

Most of the commercialized systems for local drug delivery to the lower intestine after oral administration are based on the change of pH during the gastrointestinal passage. The pH-sensitive approaches such as methacrylate/methacryl acid polymers Eudragit^®^ S and L dissolve in aqueous media at pH 6 and 7, respectively, which may be equivalent to a drug release to the distal ileum. More specifically, Eudragit^®^ P-4135F and Eudragit^®^ RS100 have been extensively investigated for the microencapsulation of 5-FU in the oral treatment of colon cancer. Moreover, Eudragit^®^ RS100 had also been successfully used for spray-coating of matrices for the treatment of colorectal carcinoma [19].

The pH-sensitive Eudragit^®^ P-4135F was used by Lamprecht *et al*. [20] to prepare 5-FU-loaded microspheres (≈ 30 % of encapsulation efficiency) by a simple oil/water emulsification process. Eudragit^®^ P-4135F, pure or in mixture with Eudragit^®^ RS100, was found to slow down drug release at pH 6.8 (less than 35 % within 6 h). However, at pH 7.4, nearly immediate release (within 30 min) was observed for pure Eudragit^®^ P-4135F, while the mixtures enabled to prolong the release slightly.

**Figure 2:**
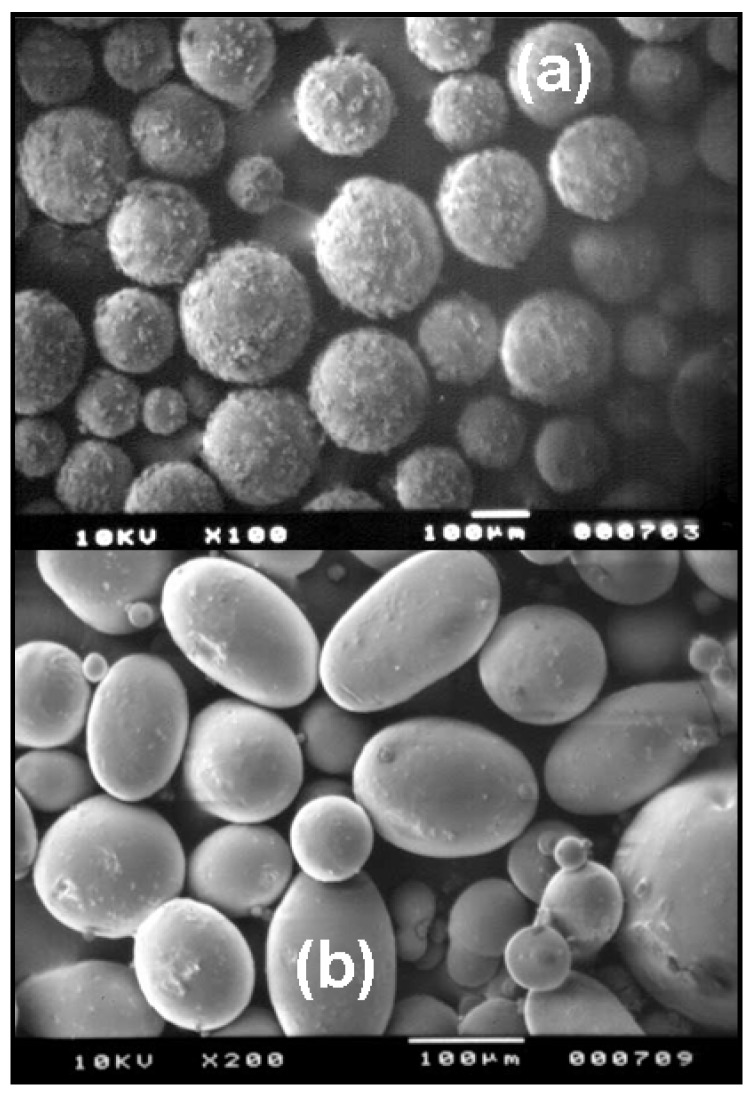
SEM images of microspheres prepared with Eudragit^®^ P-4135F (a) and Eudragit^®^ RS100 (b) using an oil/oil emulsification process. Reprinted with permission from Ref. [21]. Copyright Elsevier (2005).

These authors [21] have also used separately both Eudragit^®^ polymers to prepare microspheres by an oil/oil emulsification process (Figure 2), finding higher encapsulation rates with Eudragit^®^ RS100 (≈ 60 %) compared to Eudragit^®^ P-4135F (≈ 50 %). In few words, the preparation procedure involves the dissolution of a total polymer amount of 200 mg in 5 mL of acetone or acetone/ethanol mixtures. 50 mg of 5-FU crystals (diameter ≈ 30 μm) were suspended by ultrasonication in the polymer solution. This solution was poured into 80 mL of liquid paraffin containing 1 % (w/w) Span^®^ 80, and an oil/oilemulsion was formed by mechanical stirring. By this method, the microparticles made from Eudragit^®^ RS100 released 5-FU within 8 h. However, Eudragit^®^ P-4135F was found to maintain an undesired 5-FU release at pH 6.8 (< 25 %) within 4 h, while a nearly immediate release (within 15 min) was observed at pH 7.4. These observations are related to the high lipophilicity of Eudragit^®^ P-4135F, provoking a separation between this polymer and the drug during the preparation process. Thus, Eudragit^®^ P-4135F combined with an oil-in-oil emulsification method was observed to be very limited in use for the entrapment of this hydrophilic compound.

#### 2.1.5. Guar gum

Guar gum, a potential carrier for colon-specific drug delivery, is a polysaccharide derived from the seeds of *Cyamopsis tetragonolobus* (*Leguminosae* family). It consists of linear chains of (1→4)-β-Dmannopyranosyl units with α-D-galactopyranosyl units attached by (1→6) linkages. In pharmaceutical formulations, guar gum is used as a binder, disintegrant, suspending, thickening or stabilising agent.

A very promising tablet formulation for site-specific delivery of 5-FU to the colon was developed by Krishnaiah *et al*. [22]. Compression-coated tablets containing 80 % of guar gum were most likely to provide targeting of 5-FU for local action in the colon, since they released only ≈ 2.4 % of the drug in the physiological environment of the stomach and small intestine. Furthermore, this formulation showed no change either in physical appearance, drug content or dissolution pattern after storage at 40 °C (room humidity 75 %) for 6 months.

#### 2.1.6. Gelatin

Gelatin, a hydrophilic polymer, possesses several advantages such as being non-toxic, biocompatible, and a stabilizer with sustained release characteristics. Though the research on the applicability of gelatin particles for cancer therapy is limited, some recent studies demonstrate the potential of this drug carrier for delivering drugs to cancer due to its sustained release, biodistribution and cell internalization properties [23]. As an example, we can cite the use of this protein in the preparation of microspheres containing 5-FU [24]. The preparation procedure involves the addition of the drug solution (5-FU in water, pH 7.4) to a gelatin solution before dispersing in polymethylmethacrylate (PMMA) under mechanical stirring. One of the factors that influence the rate of drug release was the particle size of the microspheres. By varying the speed of mixing and concentration of PMMA and gelatin during preparation, microspheres of various particle sizes can be obtained (1 – 15 μm). A controlled near zero order 5-FU release profile (≈ 8 days) can be obtained by using a cocktail of microspheres in the same particle size range.

#### 2.1.7. Poly(alkylcyanoacrylates)

Poly(alkylcyanoacrylate) (PACA) nanoparticles represent an efficient form of drug carrier for drug delivery, particularly to various types of cancers. The first report on the simple and feasible technique of preparation of PACA nanoparticles by anionic polymerization appeared in 1979 [25]. After that, several studies were reported involving the influence of physicochemical and formulation factors on the formation and stability of PACA nanoparticles. One of the important advantages of PACA nanoparticles is their ability to incorporate hydrophilic drugs, providing sustained release profiles. Apart from this, PACA nanoparticles possess various advantages such as stability, biodegradability and targetability [23]. The general method of preparation of PACA nanoparticles involves the addition of an alkylcyanoacrylate monomer into an acidic aqueous solution containing stabilizers. Drugs can be incorporated into the nanoparticles by an incorporation technique involving the addition of drug before or during the polymerization, or by an adsorption technique, in which the drug is adsorbed onto the preformed nanoparticles [4, 23].

Arias *et al*. [4] have extensively studied the 5-FU loading to PACA nanoparticles (≈ 100 nm) by both loading procedures, using electrophoretic mobility and optical absorbance determinations. A positive effect of the drug concentration was observed on the 5-FU surface adsorption, and the loading efficiency increases as the hydrophobicity of the polymer, which raises with the alkyl chain length, becomes lower. The main factors determining the drug incorporation to the polymer network were the type of monomer (faster polymerization kinetics of monomers with shorter alkyl chain length, induces larger mechanical entrapping of the drug), the pH (larger encapsulation values at pH 4) and the drug concentration (positive effect). The release kinetics of 5-FU were found to be controlled by the pH of the release medium (higher at basic pHs, due to a faster polymer degradation), the type of 5-FU incorporation (faster if the drug is adsorbed onto the particles) and the type of polymer (faster from polymer matrices of shorter alkyl chain length, due to their higher degradation rate). The *in vitro* release studies showed that these 5-FU-loaded nanoparticles are suitable for sustained release.

With respect to the mechanism of incorporation of this anticancer drug into the polymer nanoparticles, it has been proposed that 5-FU is able to interfere in the initiation of the polymerization process through its nucleophilic amino groups (mainly N-1) via the formation of zwitterions and, therefore, a significant fraction of the drug is covalently bonded to the polymer chains [26, 27].

With respect to the *in vivo* efficacy of this 5-FU delivery system, Kreuter and Hartmann [28] found that the binding of 5-FU to poly(butylcyanoacrylate) nanoparticles enhanced the efficacy of this anticancer drug against Crocker sarcoma (S-180). It was observed that the polymer system yielded a prolonged persistence of 5-FU in the tumor tissue. Very promising results had also been found in patients with superficial basal cell carcinoma [29]. Briefly, ≈ 97 % of patients achieved histologically confirmed complete tumor resolution and, interestingly, this treatment did not cause significant changes both in the number of T lymphocytes and in the phytohaemagglutinin-induced DNA synthesis of T lymphocytes.

#### 2.1.8. Poly(glutaraldehyde)

The use of poly(glutaraldehyde) (PGL) nanoparticles as 5-FU carriers was studied by Mukherji *et al*. [30]. The preparation of these 5-FU-loaded nanoparticles was achieved by an aldol condensation of the monomeric glutaraldehyde under alkaline conditions. It was found that the drug-loaded (1 mg 5-FU / 7 mg PGL) was gradually released and that a higher localization of the anticancer drug was obtained in liver, lung and intestine (≈ 65 % of the initially incorporated drug).

#### 2.1.9. Polymers based on methacrylic acid

The design of hydrophilic polymer gels, the so-called hydrogels, based on derivatives of the methacrylic acid had been studied for the controlled release of 5-FU (swelling-controlled drug delivery system). As an example, poly(hydroxyethylmethacrylate-bisglycol acrylate) was used as the hydrogel network for a subcutaneous polymeric drug delivery system. The polymer-drug composite can be obtained by low-temperature radiation polymerization. Briefly, hydroxyethylmethacrylate and bisglycol acrylate comonomers are first mixed, and then 5-FU was added. The mixture is dropped into petroteum ether which is supercooled with liquid nitrogen. Polymerization was achieved by *γ*-radiation and spherical polymeric beads with a diameter of 3 mm are obtained. The suitability of this swellingcontrolled release system is supported by a biphasic drug release process that last for 20 hours [31].

Molecular imprinting is a rapidly developing technique for the preparation of polymeric materials that are capable of molecular recognition for selective preparation and chemical identification. To prepare molecularly imprinted polymers (MIPs), a functional monomer and a crosslinker are polymerized in the presence of a template molecule. The template is then extracted, leaving sites which are complementary in both shape and chemical functionality to those of the template. This polymer is capable of selectively absorbing the template species. Molecular imprinting is an efficient technique for the introduction of regions with highly specific molecular arrangements into polymer matrices. Because of the stability, predesigned selectivity and easy preparation of MIPs, they can be applied for a wide range of purposes, such as catalysis, separation and purification, detection and drug delivery. Singh *et al*. [32] had prepared 2-hydroxyethylmetacrylate-based and acrylic acid-based 5-FU imprinted hydrogels. For the synthesis of these hydrogels, *N*,*N*´-methylenebisacrylamide is used as a crosslinker, ammonium persulfate as an initiator and *N*,*N*,*N*´,*N*´-tetramethylethylenediamine as an accelerator. The recognition affinity of MIPs is increased when these are synthesized in a high concentration template (5-FU) solution. Moreover, the concentration of the crosslinker during the synthesis can play a very important role in deciding the flexibility and rigidity of MIPs. The swelling of MIPs increases with the increasing template concentration in the MIPs, which also helps in the release of the drug in a controlled manner. Puoci *et al*. [33] have also developed MIPs for 5-FU release in biological fluids by using methacrylic acid as a functional monomer and ethylene glycol dimethacrylate as crosslinking agent. It is observed that the imprinted polymers bound much more 5-FU (≈ 35 % at pH 1, and ≈ 27 % at pH 7.4) than the corresponding non-imprinted (NIPs) (≈ 6 % at pH 1 and ≈ 9 % at pH 7.4) and showed a sustained drug release. The MIPs release rate was indeed much more sustained (release of 60 % after 30 h) than that obtained from NIPs (release of 100 % after 8 h).

#### 2.1.10. Poly(α-malic acid)

Poly(malic acid) is a water-soluble biodegradable and non-antigenetic lactide type polyester having pendant modifiable carboxylic acid groups that can be used as a bioresorbable polymer in the design of drug carriers. Ohya *et al*. [34] had prepared poly(α-malic acid)-5-FU-saccharide conjugates by attaching 5-FU and some kinds of saccharide residues (galactosamine, glucosamine, *N*-acetyl-D-glucosamine, mannose and mannosamine) to poly(α-malic acid). The obtained conjugates showed significant survival effects against P-388 leukemia mice and did not display an acute toxicity in the dose range studied (200-800 mg/Kg). The poly(α-malic acid)-5-FU-galactosamine conjugate tended to exhibit a higher growth-inhibitory effect against hepatoma cells than the other conjugates. An active targeting process was described for this carrier which is thought to be achieved by the appearance of a cell specific affinity of the conjugate for hepatoma cells via galactose-receptor mediated endocytosis.

Finally, the preparation of poly(α-malic acid) conjugates had also been carried out for the controlled delivery of 5-FU derivatives with very promising results in mice with P-388 lymphocytic leukemia [35]. It was observed that when the unreacted pendent carboxylic acid groups in the poly(α-malic acid)-5-FU conjugates were masked by methyl groups, a prolonged half-life was exhibited by the conjugates, no acute toxicity was observed for doses between 200 and 800 mg/Kg and a higher antitumor activity was determined (higher mice survival values).

#### 2.1.11. Poly(methilidene malonate 2.1.2)

Poly(methylidene malonate 2.1.2) (PMM 2.1.2) is a novel biocompatible material characterized by a long degradation time, due to its chemical structure, which could be advantageous in the treatment of long evolving pathologies, avoiding repetition of stereotactic injections.

5-FU-loaded PMM 2.1.2 microspheres (40 – 50 μm; loading efficiency ≈ 25 %) were formulated by Fournier et al. [36] by an emulsion-extraction procedure, and evaluated on a C-6 glioma model. This technique is based on the formation of an oil-in-water emulsion under controlled stirring. Briefly, 5-FU crystals are ground and 300 mg are suspended in 6 mL of methylene chloride under vigorous stirring. The polymer (300 mg) is then dissolved in the organic solvent under magnetic stirring. The suspension is driven to a temperature lower than 5 °C, and an emulsion was obtained by pouring the organic phase into a polyvinyl alcohol aqueous phase (200 mL, 2 % w/v, 2 °C) under mechanical stirring. 300 mL of deionized water (4 °C) are then added to the emulsion, allowing the formation of microspheres, which resulted from the solvent extraction. These microspheres showed a biphasic release profile (60 % during day 1, 40 % during 47 days) and their efficacy was demonstrated by an improvement in the survival of C-6 glioma-bearing animals, and also by a decrease in the tumor burden. Briefly, the median survival of the treated group (34.3 ± 9.2 days; *n* = 4) was significantly longer than the controls (20.6 ± 1.7; *n* = 5; *P* = 0.02). These increase in life span appeared to be linked to a limitation of C-6 glioma cell proliferation as shown by the *in vivo* growth curves measured by using MRI, where a 4 – 15-day long lag was observed.

The long degradation time of PMM 2.1.2, slower than poly(lactide-co-glycolide), is attributed to the chemical structure of this polymer (ester bonds in the side chains only), and assures a much more prolonged drug release in a specific area of the brain [37].

#### 2.1.12. Polyacrylamide

5-FU-loaded polyacrylamide (pAAm) particles (2 – 3.5 μm) crosslinked with *N*,*N*-methylenebisacrylamide (NNMBA) or ethylene glycol dimethacrylate (EGDMA) are prepared in a water–methanol medium by the dispersion polymerization technique. Both encapsulation efficiency and release patterns depend on the nature of the crosslinking agent, the amount of crosslinking agent used and the percentage of drug loaded. Higher encapsulation efficiency (≈ 80 %) is achieved for particles crosslinked with the hydrophilic NNMBA. This is attributed to a lesser interaction of the hydrophilic drug with the hydrophobic crosslinking agent. However, a prolonged drug release (up to 12 h) is observed when EGDMA is used, due to a lesser swelling capability of the particles obtained [38].

These authors had also prepared 5-FU-loaded poly(acrylamide-methylmethacrylate) copolymeric core-shell microspheres (≈ 30 μm) crosslinked with NNMBA, by free radical emulsion polymerization of the corresponding monomers [39]. Briefly, sodium laurylsulfate (1 g) and sodium hydrogen phosphate (100 mg) are dissolved in water (80 mL) in a three-necked round bottom flask equipped with a mechanical stirrer, a condenser and a gas inlet to maintain the inert nitrogen atmosphere. The solution is stirred until it became clear and potassium persulfate (100 mg) is added. AAm:MMA (1:1), NNMBA (1 %) and 5-FU (15 %) are dissolved separately in water (20 mL). Both aqueous solutions are mixed dropwise and the reaction is continued for 8 h at 70 °C. Then, the reaction mixture is taken out and added drop-wise to a 1 % calcium chloride solution to break the emulsion. Particles are then isolated by simple centrifugation. The *in vitro* sustained drug release (≈ 24 h) indicated that particle size and release kinetics depend upon the copolymer composition, the percentage of crosslinking agent used and the amount of 5-FU loaded by the microspheres.

The analysis of the 5-FU release kinetics carried out by the authors hypothesized that, due to the interaction between the anticancer drug and the core-shell microspheres, the release from the matrix occurred by Fickian diffusion. It was observed a faster release rate in formulations with higher 5-FU loading values, as it is usually the case [4]. However, a slower drug release rate was determined in formulations with lower 5-FU encapsulation. The latter could be probably due to the availability of more free-void spaces through which lesser 5-FU molecules could diffuse. With respect to the effect of the concentration used of crosslinking agent (NNMBA), the release rate was faster in formulations containing lower amounts of NNMBA (1 %), whereas the release was quite slower at higher concentrations (3 %). This was explained taking into account that at higher NNMBA concentrations, the polymeric chains will become rigid (due to contraction of microvoids) and it will induce a decrease in the cumulative drug release. Finally, as the copolymer composition was determined by the amount of crosslinking agent included in the formulations (the monomer concentration did not change), all the observations about the influence of the NNMBA concentration on the release kinetics were true with respect to the influence of the copolymer composition on 5-FU release.

#### 2.1.13. Poly(ortho-ester)s (POE)

Glaucoma filtering surgery consists in the production of a filtration fistula to facilitate the outflow of aqueous humor from the anterior chamber and to reduce the intraocular pressure. However, a wound healing process can seal the surgical site and result in failure of the operation. Fibroblast proliferation plays an important role in this wound healing and scarring process. Various pharmacological agents such as antimetabolites and anti-inflammatory steroids have been shown to effectively inhibit the growth of fibroblast *in vitro*. 5-FU and dexamethasone are frequently administered together post-operatively due to their anti-fibroblastic and anti-inflammatory properties, respectively. Zignani *et al*. [40] had developed a viscous hydrophobic POE as a biocompatible and biodegradable sustained release system for both drugs. It was observed that, due to its basicity, the addition of dexamethasone sodium phosphate (DEX-P) stabilized the polymer and prolonged 5-FU *in vitro* release up to 4 days. Both therapeutic agents were released concomitantly, according to a linear profile from this carrier. The presence of 5-FU only slightly affected the overall subconjunctival tolerance of POE in rabbits, whereas the addition of DEX-P markedly improved POE tolerance by reducing the hyperemia of the conjunctiva to a minimal grade. This is a promising way to treat glaucoma filtration surgery failure after subconjunctival administration (intravitreally for proliferative vitreoretinopathy or subretinally for choroidal neovascularization) [40, 41].

The synthesis procedure involves a transesterification reaction between 1,2,6-hexanetriol and trimethyl orthoacetate. The reaction, catalyzed by *p*-toluenesulfonic acid, can be carried out by distilling the two precursors in cyclohexane under anhydrous conditions. Finally, POE is purified by a precipitation procedure and dried under vacuum at room temperature [40, 41].

Viscous poly(ortho-ester)s (POE) can also be synthesized by a transesterification reaction between a substituted orthoacetate (trimethylorthoacetate) and a triol (1,2,6-hexanetriol), and used as injectable bioerodible polymers for controlled release of 5-FU in glaucoma filtering surgery. It was observed that by replacing the 1,2,6-hexanetriol with 1,2,10-decanetriol, a higher molecular weight aliphatic triol, a less viscous and more hydrophobic polymer is obtained. Due to the decreased viscosity, the polymer can be easily used for injectable applications and, due to the increased hydrophobicity, the release rate of 5-FU is slowed down compared to the former POE. This modification in the formulation allows obtaining a 5-FU-loaded polymer with adequate properties in glaucoma filtering surgery [42].

Another family of POEs can be prepared by a condensation of diols and the diketene acetal 3,9-diethylidene-2,4,8,10-tetraoxaspiro[5.5]undecane, which also incorporates into the polymer backbone a short lactoyl-lactyl or glycoyl-glycyl segment. Therefore, this polymer differs from the previously described polymers in that it incorporates a short segment of a latent acid in the polymer backbone. Bioerodible 5-FU delivery systems based on this polymer allows sterilization, and a good control over erosion rates (5-FU sustained release over 30 days) and mechanical properties can be achieved by appropriate selection of the diols used in the synthesis. Moreover, implantation studies in rats have shown that the polymer erodes to completion and that is not overtly toxic [43]. This family of polymers had been successfully tested in a clinical trial showing an increment in the efficacy of the treatment and good tolerance [44].

#### 2.1.14. Poly(D,L-lactide) (PLA) and poly(D,L-lactide-co-glycolide) (PLGA)

Of the various polymers used for the preparation of nanoparticles, poly(D,L-lactide-co-glycolide) (PLGA) and poly(D,L-lactide) (PLA) are highly preferred due to their biocompatibility and biodegradability, and are FDA-approved polymers for human use. Upon administration, these polymers degrade by hydrolysis into lactic and glycolic acids, which are eventually removed from the body by the citric acid cycle. Emulsification followed by solvent evaporation is a widely applied technique for the preparation of PLA and PLGA nanoparticles, employing polyvinyl alcohol (PVA) as a stabilizer. Generally, this method involved the emulsification of an organic polymer solution into an aqueous phase containing PVA, followed by the evaporation of the organic solvent to result in smaller particles with uniform size distribution. The size of the nanoparticles decreased with an increase in the PVA concentration used for the particle preparation. The amount of residual PVA in the final nanoparticles is important because it influences the physicochemical properties and biological behavior of the nanoparticles. PLGA nanoparticles are widely studied for delivering anticancer agents to tumors [23].

Poly(D,L-lactide) (PLA) polymer is under investigation for the design of novel block copolymer loaded with 5-FU. As an example, Lo *et al*. [45] had developed thermo-responsive, pH-responsive and biodegradable nanoparticles comprised of poly(D,L-lactide)-graft-poly(*N*-isopropylacrylamide-comethacrylic acid) by grafting biodegradable PLA onto *N*-isopropylacrylamide and methacrylic acid. A core-shell type nano-structure is obtained with a hydrophilic outer shell and a hydrophobic inner core, which exhibited a phase transition temperature above 37 °C suitable for biomedical applications. Thus, upon heating above the phase transition temperature, the structure of the copolymer causes leakages from outer shell, copolymer aggregation and collapsed. A sustained 5-FU release (biphasic profile) during 4 days is developed, strongly controlled by the pH.

Different strategies had been investigated for the preparation of 5-FU-loaded PLA particles with an extensive biodistribution. One of the most commonly followed is the preparation of PLA-PEG-PLA triblock copolymers by ring opening polymerization [46, 47]. However, very little control over the release properties is obtained, a testing to a relatively inefficient “core” trapping for the drug (≈ 15 %).

In order to increase 5-FU loading to PLA nanoparticles diverse synthesis procedures had been investigated. For example, a modified oil-in-oil emulsion solvent evaporation technique had been successfully developed [48]. In this technique, the disperse phase is a drug:polymer solution using a solvent mixture of *N*,*N*-dimethylformamide (DMF) and acetonitrile, and the continuous phase is a liquid paraffin containing 1 – 10 % Span^®^ 80. By introducing a 25 % (v/v) of DMF into the inner oil phase, microspheres with high 5-FU entrapment efficiency (≈ 80 %) and an ameliorated burst release effect are prepared. *In vitro* drug release tests showed a burst 5-FU release followed by a sustained release over 50 days.

PLGA particles loaded with 5-FU can also be obtained by a water-in-oil-in-water emulsion solvent evaporation technique [49], where an increase in the volume of the internal phase of the primary emulsion or in the volume of the external phase of the secondary emulsion, resulted in a decrease in the encapsulation efficiency and in an increase in the particle size. Another preparation procedure used in the synthesis of 5-FU-loaded PLGA (PLA) particles is the spray-drying technique [50], which allows the formation of small size microspheres (≈ 1.5 μm) with a smooth and slightly porous surface, and a significant entrapment efficiency (50 – 75 %). A biphasic release profile is observed for these microparticles (20 % after 1 h) that last for ≈ 12 h. The PLGA microspheres prepared by the spraydrying technique are degraded (after a subcutaneous injection in rats) and they are not detected at the injection site after 2 – 3 months [51]. In comparison to the free anticancer drug, the mean residence time (MRT) in plasma of 5-FU released from PLGA significantly increases up to ≈ 80 – 140 hours (≈ 1 – 3 hours in the case of free drug). Moreover, the drug was continuously detected in plasma between 9 and 14 days.

The use of the nanoprecipitation-solvent displacement technique assures the formation of drugloaded PLGA particles in the nanosize range (160 – 250 nm), characterized by a high entrapment efficiciency (≈ 80 %) [52]. On the other hand, the synthesis of PLGA nanoparticles loaded with 5-FU (200 – 300 nm) by the emulsion-solvent diffusion technique reduce the drug loading to only 3 % [53].

As it was observed in other polymeric devices [4], the release rate of 5-FU was increased with the increase of 5-FU loading. Nevertheless, the 5-FU release profiles followed a near first order release kinetic independently of the drug loading [54,55,56]. PLGA nanoparticles were characterized by high 5-FU loading properties in comparison to other widely-used carriers such as poly(ε-caprolactone), Eudragit^®^ or poly(butylcyanoacrylate) [54]. However, in this study the best sustained release properties are developed by Eudragit^®^ particles.

A triphasic release process had been described for 5-FU from PLGA particles [57]. The initial drug release phase is primarily controlled by 5-FU diffusion and the limited solubility of the drug. In the second 5-FU release phase, also polymer degradation becomes important: the increase in the length of the diffusion pathways with time (which should lead to a decrease in the release rate) is compensated by an increase in drug mobility (which results from the decrease in the average polymer molecular weight and, thus, increased macromolecular mobilities). The final rapid drug release phase can be attributed to the disintegration of the microparticles (as soon as the average polymer molecular weight reaches a certain critical threshold value, the polymeric network looses its mechanical stability), resulting in significantly shortened diffusion pathways.

The size of the biodegradable particles can determine the 5-FU release from PLGA. It has been observed that the initial drug loading significantly increased with increasing radius of the drug carrier. However, the absolute 5-FU release rate also increases with the size. This can be explained if we take into account that large microparticles became more porous during drug release than small particles, leading to higher apparent diffusitives and drug transport rates. This effect overcompensate the effect of the increasing diffusion pathways with increasing microparticles radius, resulting in increased drug release rates with increasing device dimension [58].

The effect of the type of release medium clearly also determine the 5-FU release from PLGA microparticles [57]. Very different drug release patterns (including mono-, bi- and tri-phasic ones) can be observed depending on the osmolarity, pH and temperature of the release medium. It was observed that the drug release rate decreased with increasing osmolarity of the release medium, probably due to a decrease in the water uptake rate and extent of the system. With decreasing water content, the mobility of 5-FU decreases and, thus, the release rate decreases. Furthermore, as the pH of the release medium is increased, the degradation rate of the polymer becomes slower. Finally, the release rate significantly increased as the temperature rises from 37 to 65 °C, probably due to the increased mobility of the polymer chains and drug molecules at elevated temperature, resulting in higher diffusion rates.

Interestingly, the application of *γ*-irradiation to the microspheres for sterilization also determines the 5-FU diffusion from the polymer. A quantitative relationship between the applied *γ*-irradiation dose and the resulting initial drug diffusion coefficient within the systems can be established. Using these relationships it is possible to predict the resulting drug release kinetics for arbitrary irradiation doses [59, 60].

Several studies are ongoing about the use of PLGA-based microspheres to deliver 5-FU to the central nervous system. In particular, 5-FU-loaded PLGA microspheres have been developed for stereotactic intracerebral implantation [61]. Preliminary experiments on C-6 glioma-bearing rats have shown very promising results: the median survival time might be doubled [62]. Eight patients of a phase I–II pilot study, scored for high-grade glioma, underwent surgical removal before 5-FU-PLGA microspheres were implanted in the resection bed. Eighteen months after the trial began, results were very encouraging as far as survival, welfare and future applications were fore-cast [63]. Compared with monolithic implants such as Gliadel^®^, the microspheres allowed a higher adaptability of the treatment to the surgical act [64]. Indeed, depending on how deep and how distant from each other microspheres are deposited, the area covered by the drug can be controlled. The microsphere fate and 5-FU-diffusion area from these particles were studied by Roullin *et al*. [65], finding that an important back flow occurs in healthy rats, whereas the microspheres remain at the site of administration in C-6 glioma-bearing rats. It was also determined that drug diffusion is limited to the vicinity of the implantation site. In a more recent study, it was probed that 5-FU-loaded PLGA implanted by stereotaxy into the tumor are well-tolerated (no episodes of edema or hematologic complications occurred) and are efficient systems for drug delivery into brain tumors. This method may have future applications in the treatment of patients with other malignancies [66]. A phase I/II clinical trial has been carried out in patients with newly diagnosed glioblastomas, showing very interesting results about the potentialities of these 5-FU-loaded microspheres when intracranially implanted [67].

PLGA matrices had also been proposed for the treatment of peritoneal carcinomatosis, showing a slowly 5-FU release during three weeks. Intraperitoneal 5-FU matrices distributed higher drug concentrations to the intraperitoneal tissues, for a longer period and with lower plasma concentrations, than did the aqueous 5-FU solutions in rats. It was concluded that the therapeutic effects were enhanced and the lethal toxicity was reduced to less than a half that in 5-FU solutions [68]. Moreover, pharmacokinetic analysis showed that the area under the curve (AUC) was significantly greater in the intraperitoneal tissues (omentum and mesentery) that in other tissues [69].

Antisense oligonucleotides (AODNs) can selectively inhibit oncogene expression by Watson–Crick hybridisation to target mRNA and are being increasingly considered for use in combination with conventional drugs for potential anticancer therapy. Combination therapy of AODNs and cytotoxic agents (such as 5-FU) using biodegradable polymeric delivery systems potentially offers several advantages including site-specific or organ-directed targeting, protection from digesting enzymes, and improved pharmacokinetics/pharmacodynamics resulting from sustained delivery of the entrapped drugs. For these reasons, PLGA microspheres formulations had been investigated for co-delivery of these agents. Using a double emulsion method for preparing the PLGA microspheres suitable entrapment and sustained release over 35 days was obtained. Release of co-entrapped AODN and 5-FU from single PLGA microsphere formulations appeared to be biphasic and significantly slower compared to those obtained with separate formulations. Electrophoretic mobility shift assays suggested that this might be, in part, due to an interaction of 5-FU with the oligodeoxynucleotide. Thus, coadministration of PLGA microsphere formulations of AODNs and 5-FU, at appropriate mass ratios, appears worthy of further investigation for the potential co-delivery of these anti-cancer agents *in vivo* [70].

Co-encapsulation of 5-FU in PLGA microspheres (2.5 μm) was also investigated with the anticancer drug paclitaxel. The encapsulation efficiency of 5-FU was increased from 20 % to 30 % when the drug was encapsulated with paclitaxel. This could be in part due to the formation of a physical blend between both drugs. Overall results of this investigation demonstrated that the encapsulation of both drugs in the microspheres enhances the cytotoxicity in a more controlled manner, compared to that of free drugs [71].

The formulation of 5-FU-loaded PLGA particles is also intended for other delivery routes such as oral, ocular or pulmonar. In the former case [72], it was observed that a single oral administration to rats of the loaded-nanoparticles produced a statistically higher bioavailability than a 5-FU solution administered as a negative control. With respect to the ocular administration of these systems, preliminary animal studies indicated that 5-FU-loaded microparticles showed no ocular toxicity and no significant inflammatory response in rabbits for 2 months and, thus, it might be potentially useful as a complement drug system in glaucoma filtration surgery [73, 74]. For the pulmonary route, the aerodynamic particle size should be less than 1 μm to minimize the deposition of the particles in the nasopharyngeal area. Moreover, the duration of the delivery should be extended to allow daily administration. Hitzman *et al*. [75] had developed 5-FU-loaded liposomes, microspheres (PLA and PLGA) and lipid-coated nanoparticles [made of a poly(glutamic acid) core and a tripalmitin/cetyl alcohol shell] that fulfill both requisites and, therefore, may be suitable as an inhalation delivery system for adjuvant therapy of lung cancer.

#### 2.1.15. Dendrimers

Dendritic polymers (or dendrimers) are hyperbranched, uniformly distributed structures, having definite molecular weight, shape, size and host-guest entrapment properties. They are characterized by a globular, highly branched and regular repeated molecular architecture that is constructed via stepwise procedures. In comparison with other classical polymers and oligomers, dendrimers have some unique characteristics. Because dendrimers are not synthesized by ordinary polymerization, they are theoretically monodisperse. Dendrimers have many end functional groups that can be linked with other chemical moieties. Thus the surface properties of dendrimers can be greatly modified. Furthermore, considering the outer layer, a variety of biologically active agents can be incorporated into dendrimers to form conjugates, and act as an interesting kind of drug carriers, or carrier for gene transfer.

With the aim of developing dendrimer-5-FU conjugates, Zhuo *et al*. [76] prepared dendritic polymers with a core of 1,4,7,10-tetraazacyclododecane. Briefly, this core was allowed to react with methyl acrylate through a Michael addition reaction and was then amidated with ethylenediamine. Repeating the two steps led to controlled molecular weight increasing and branching on the molecular level and produced four direction poly(amide-amine) dendrimers. This acetylated dendrimers were then reacted with 1-bromoacetyl-5-fluorouracil to form a 5-FU dendrimer carrier that reduced the drug toxicity due to a slow release (≈ 10 days).

Bhadra *et al*. [77] focussed their investigation in the design of PEGylated polyamidoamine (PAMAM) dendrimers. Successive and exhaustive amidation reactions can be followed to synthesize 4.0G PAMAM dendrimers, using ethylenediamine as core and methyl methacrylate as propagating agent. The dendrimer are PEGylated by using *N*-hydroxysuccinimide-activated carboxymethyl MPEG-5000. The PEGylation of the system can increase their drug-loading capacity, and reduce their drug release rate and haemolytic toxicity.

### 2.2. Hydrogels

Hydrogels (hydrophilic polymer gels) are three-dimensional polymeric networks composed of a polymer backbone, water and crosslinking agents, which swell quickly by imbibing a large amount of water or shrink in response to changes in their external environment. These modifications can be induced by changing the surrounding pH, temperature, ionic strength and electrostimulus.

Gellable thermosensitive poly(*N*-isopropylacrylamide-co-acrylamide) (PNIP/AAm) nanogel aqueous dispersions can be prepared through a precipitation-polymerization procedure. The prepared PNIP/AAm nanogel particles loaded with 5-FU show good thermosensitivity and drug entrapment efficiency (≈ 30 %). These hydrogels are in swollen status below the volume phase transition temperature (VPTT) and in shrunken status above the VPTT. Taking the advantage of temperatureresponsivity of the PNIP/AAm, drug loading and release ability could be intelligently triggered by a temperature change [78].

The 5-FU-loaded hydrogels with sustained release characteristics can also be based on copolymers of *N*-(2-hydroxypropyl)methacrylamide and *N*,*O*-dimethacryloylhydroxylamine [79]. Ravichandran *et al*. [80] had also prepared pH sensitive, biocompatible hydrogels as 5-FU delivery systems. These hydrogel are poly[*N*-vinyl pyrrolidone-acrylic acid]-polyethylene glycol interpolymer type systems that can be easily prepared by free radical polymerization. In the synthesis procedure, azobisisobutyronitrile and *N*,*N*´´-methylene bisacrylamide are employed as initiator of the polymerization and crosslinking agents, respectively. The slow 5-FU release from these structures (biphasic profile) may be attributed to the very slow hydrolytic degradation of the polymer matrix.

5-FU had also been entrapped in a collagen-poly(hydroxyethylmethacrylate) hydrogel matrix to achieve controlled release properties (zero-order kinetics for 10 days) [81]. Another possibility is to load 5-FU into a poly(*N*-isopropylacrylamide) (PNIPA) hydrogel that will subsequently be carefully enveloped in a dialysis bag to form a novel thermo-responsive drug delivery system [82]. Compared to the negative drug release pattern of the conventional PNIPA, this novel smart-drug delivery system can give a positive drug release pattern, i.e. rapid 5-FU release rate at an increased temperature (> 25 °C) and slow release rate at a decreased temperature (≤ 25 °C).

Finally, Woolfson *et al*. [83] designed a bioadhesive cervical patch containing 5-FU for the treatment of cervical intraephitelial neoplasia. The patch present a bilaminar design with a drug-loaded bioadhesive film cast from a gel containing 2 % (w/w) Carbopol^®^ 981 plasticised with 1 % (w/w) glycerine. The casting solvent is ethanol/water 30:70, chosen to give a non-fissuring film. It was probed that, despite the hydrophilic nature of 5-FU, substantial drug release through human cervical tissue samples is achieved during ≈ 20 h.

### 2.3. Vesicular systems: liposomes and niosomes

#### 2.3.1. Liposomes

Liposomes are biocompatible, biodegradable microvesicular systems consisting of at least one phospholipid bilayer. They can encapsulate water-soluble compounds in internal aqueous compartments (whether in their core or between lamellae) and/or intercalate lipophilic molecules in their bilayer(s). Liposomes can be prepared as sterile, pyrogen-free suspensions in submicron diameters in order to facilitate intravenous injection. The superiority of liposomes as drug carriers is widely recognized and great advances in the liposome field have resulted in the development of some approved liposomal products. Several synthesis procedures had been studied in order to encapsulate 5-FU in liposomes consisting in unilamellar, plurilamellar or multilamellar vesicles made of diverse types of phospholipids. Furthermore, 5-FU prodrugs such as alkylcabamoyl derivatives and N_3_-O-toluyl-fluorouracil had also been successfully incorporated to liposomes [84, 85].

Elorza *et al*. [86] studied the entrapment of 5-FU in unilamellar liposomes prepared by the freethawing extrusion technique (FATVET) and the reverse-phase evaporation method (REV) from natural (bovine brain) sphingomyelin and synthetic distearoylphosphatidylcholine. REV vesicles (200 nm) were found to entrap roughly double amounts of drug than did extruded liposomes (100 nm). The rate of *in vitro* drug release from the liposomes was found to be dependent of the bilayer composition and of the method used to prepare the vesicles. The results suggested that 5-FU release is kinetically controlled by an interfacial process seemingly dependent on the surface activity of the drug. Also, the physical state of the bilayer determines the retention capacity of the vesicles.

5-FU-loaded liposomes can also be prepared by a method based on the lyophilization of double emulsions containing disaccharides as lyoprotectans in both the inner and outer aqueous phase [87]. Using various phospholipids or mixtures of lipids as emulsifiers, the double emulsions can be prepared by a two-step emulsification, including hydrophilic agents in the inner aqueous phase or lipophilic agents in the oil phase. Then, the double emulsions are lyophilized after sterilization by passing them through a 0.22 μm pore filter. Rehydration of the lyophilized products results in liposomes with a relatively low 5-FU encapsulation efficiency (≈ 20 %) and a size below 200 nm. The liposomes are unilamellar and can be reconstituted just before use by rehydration of the lyophilized products which are relatively stable.

The microencapsulation vesicle method is a liposome preparation technique in which liposomes of homogeneous particle sizes and low 5-FU encapsulation efficiency values (≈ 15 %) are formed through a two-step emulsification and dispersion with mechanical agitation [88].

Lipid mixtures containing dipalmitoylphosphatidylserine seemed to be the best for *in vivo* 5-FU delivery due to their proved better encapsulation efficiency, serum and storage stability, and fusogenic properties (an important prerequisite for *in vivo* liposome-cell interaction) than other phospholipids [89]. The presence of cholesterol in the liposome composition is an important factor thereby ensuring serum and storage stability of the vesicular system. Moreover, it was recommended to lyophilize the liposomes with saccharose as cryoprotectant, in order to increase their stability and make them less permeable to the encapsulated drug (80 % of 5-FU retention after lyophilization/rehydration). Finally, no effect on the particle size is observed after freeze-drying liposomes containing this cryoprotectant [90, 91].

Important advances were also achieved in the field of active targeting of brain tumors using nanocarriers such as liposomes. Transferrin (Tf) receptors are overexpressed on the brain capillary endothelium and at the surface of proliferating cells such as brain tumor cells, especially glioblastoma multiforme. Tf-liposomes used for the delivery of the antimetabolic drug 5-FU to the brain had demonstrated *in vivo* that their accumulation within brain capillary endothelial cells is 13-fold higher than that of non-modified liposomes, suggesting the likelihood of an enhanced 5-FU brain uptake [92].

#### 2.3.2. Niosomes

Niosomes are formed by the self-assembly of non-ionic amphiphiles in aqueous media resulting in closed bilayer structures. The greater stability, lower cost of surfactants and less storage problems have prompted for the exploitation of these vesicles as an alternative to phospholipid ones. Niosomes based on a novel crown ether amphiphile [1,16-hexadecanoyl-bis-(2-aminomethyl)-18-crown-6 (Bola A-16)] with an average size of 200 – 400 nm, had been proposed as a topical delivery system for 5-FU in the treatment of different forms of skin cancers. It was found a high loading efficiency (≈ 40 %) and a biphasic 5-FU release pattern (sustained release). 5-FU-loaded bola-niosomes provided an increase of the drug penetration in the skin of ≈ 8-folds and, therefore, showed an improvement of the cytotoxic effect with respect to the free drug [93, 94].

### 2.4. Magnetic drug delivery systems

Magnetic drug delivery systems are constituted by particles capable of carrying the desired amount of drug to the required target and releasing it at a controlled rate. Magnetic colloidal particles could in principle be a suitable drug vehicle, because of their magnetic-field responsiveness: an applied magnetic field could drive the particle to a desired location and keep it there for a given period of time. Since particles do not spread in the general circulation their recognition and elimination by the mononuclear phagocyte system may take a long time. 

Magnetic particles alone, however, are not best suited as drug vehicles, because of limitations in the control of the amount of drug they carry and the rate at which they can release it. Unlike inorganic magnetic particles, biodegradable polymers can release previously absorbed drugs at a rate determined by their (essentially harmless) degradation. A mixed particle composed of a magnetic nucleus and a biodegradable polymer shell could take advantage of the properties of its two components. The magnetic core could make it possible to direct the particles to the specified location (and keep them there) by means of a magnetic field. This, in turn, would reduce the side effects of the drug because its levels in the general circulation are lowered. Finally, the polymeric shell would have the role of transporting the drug and releasing it during its biodegradation [5].

**Figure 3:**
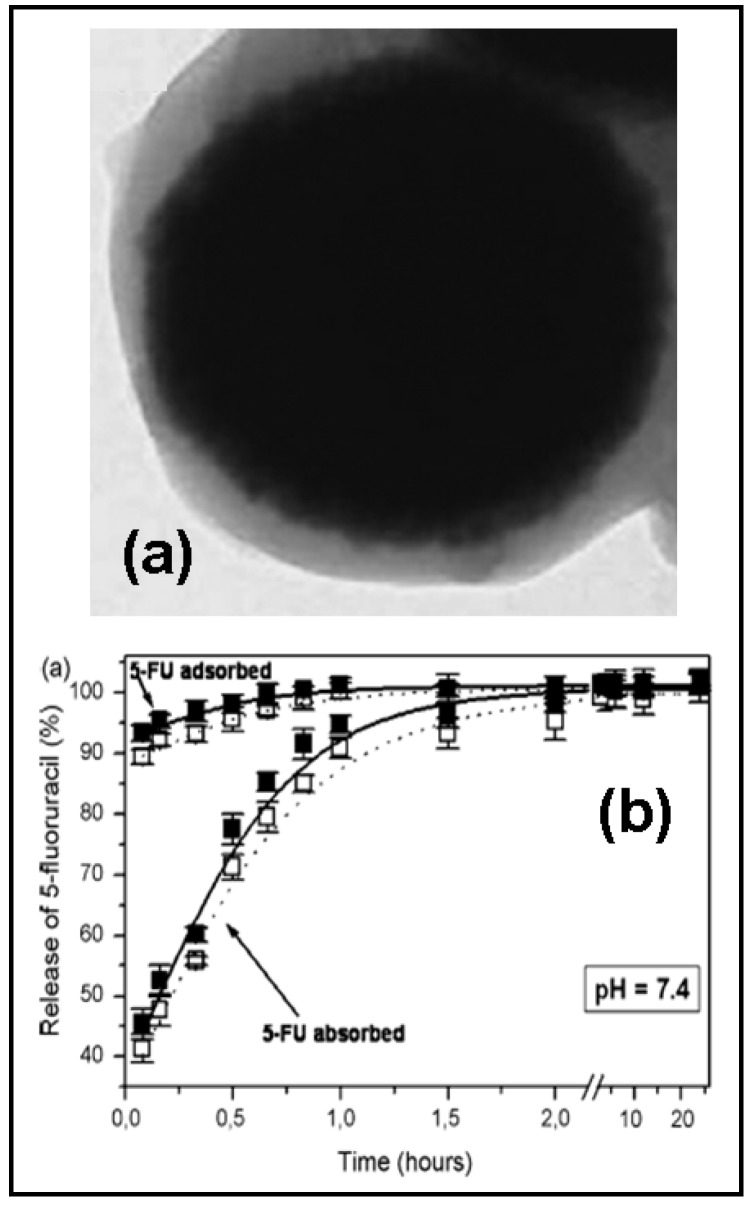
Transmission electron microscopy picture of carbonyl iron/poly(butylcyanoacrylate) (core/shell) nanoparticles (a) and *in vitro* 5-FU release (%) as a function of the incubation time (b). Reprinted with permission from Ref. [96]. Copyright Elsevier (2008).

Actually, poly(alkylcyanoacrylates) are the most investigated polymers for the design of polymeric shells in the development of 5-FU magnetic carriers, while the magnetic nuclei are made of iron oxides (magnetite) or carbonyl iron (Figure 3a) [95,96,97]. The anionic polymerization procedure, used to obtain poly(alkylcyanoacrylate) nanoparticles for drug delivery, is followed in the synthesis of the composite particles, except that the polymerization medium is a carbonyl iron or magnetite suspension. 

The entrapment of 5-FU in the polymeric network had shown best drug-loading results than surface adsorption, as was determined by spectrophotometric and electrophoretic measurements. Both pH and drug concentration were found to be the main key-role parameters in 5-FU loading. The release profile was found to be biphasic, since the drug adsorbed on the surface was released rather rapidly (close to 100 % in one hour), whereas the drug incorporated in the polymer matrix required between 10 and 20 hours to be fully released (Figure 3b). The kinetics of the drug release from the core/shell particles were mainly controlled by the pH of the release medium, the type of drug incorporation, and the amount of drug loaded.

Viroonchatapan *et al*. [98] prepared 5-FU-loaded thermosensitive magnetoliposomes by reversephase evaporation, in which the drug release can be executed several times in response to an electromagnetic field-generated heat (hyperthermia).

### 2.5. Lipoproteins

An attempt was made to improve the pharmacokinetic behavior of 5-FU by incorporating it into a lipoprotein imitating synthetic nanocarrier “supramolecular biovector (SMBV)” [99]. The polysaccharide core of SMBVs can be prepared by an ionotropic gelation technique in which polyguluronate units are cross-linked in alginate molecules with calcium ions to form the so-called “egg-box structure”. It was concluded that the enhanced delivery of the anticancer drug to lymphatics and the improvement in its half-life render SMBVs useful for the control of metastasis and tumor growth.

5-FU had also been assayed with high density lipoproteins (HDL), low density lipoproteins (LDL) and very low density lipoproteins (VLDL), and significant drug loading values were achieved [100]. It was observed that the anticancer drug remains stable in the lipoproteins and that the cytotoxicity of the 5-FU-loaded lipoproteins was significantly lower in comparison to the free drug.

### 2.6. Clay minerals and anionic clays

Montmorillonite, a bioinert clay mineral, consists of hydrated aluminum silicates with fine grains and large spaces between the layers. It has a great capability to intercalate large molecules into the interlayer space of the plane, due to a more open inter-space in basic conditions, especially for pH values > 11. The structure of montmorillonite is an octahedral laminated sheet, sandwiched between tetrahedral silicate layers. Due to these characteristics, attempts had been made to intercalate 5-FU into its interlayer under optimum conditions (soaking time, temperature, pH value, initial 5-FU concentration, etc.) in order to develop a novel composite 5-FU delivery system with the ability of releasing the antimetabolite drug in situ to treat colorectal cancer [101]. 5-FU has been confirmed to successfully intercalate into the interlayers of montmorillonite both by free surface adsorption, and sodium and OH replacement.

Owing to the rich intercalation chemistry, layered double hydroxides (LDH, anionic clays or hydrotalcite-like compounds) have extensive applications as catalysts, catalyst precursors and supports, adsorbents, optical and electric functional materials, and flame retardants and polymer stabilizers. Recently, particular attention has been focused on LDH-based controlled release systems. Wang et al. [102] had built up, through a calcination-restructure method, a biocompatible chargeneutral Mg_2_Al LDH system for the vehiculization of 5-FU. It was found that the interlayer arrangement of 5-FU depends on various patterns of aging treatment and different swelling solvents. The release studies showed that a rapid 5-FU release from LDH during the first 40 min (≈ 65 % at pH 4, and ≈ 50 % at pH 7) was followed by a slightly sustained one, being the total amount of anticancer drug released from the hybrid material ≈ 85 % at pH 4 and ≈ 75 % at pH 7, after ≈ 2.5 hours. The observed differences in the release patterns between both pHs were attributed to different release mechanisms. At pH 7, the drug release mechanism was thought to be based on an ion-exchange process between 5-FU anions pillared in the lamella host and phosphate anions of the buffer solution. On the contrary, at pH 4 the LDH could start a dissolution process so that the faster 5-FU release occurred through ion-exchange and the removal of inorganic host.

### 2.7. Metals

The binding of 5-FU to colloidal gold via complexation through the -NH group had been extensively studied [103]. From the data obtained in the biological assays, it is inferred that the combination of the anticancer drug with gold nanoparticles have appreciably antibacterial and antifungal activity against gram-negative bacteria. The combination of gold with 5-FU results in a more potent complex, compared to the individual parts. The nature of this interaction, as evidenced from fluorescence and electrochemical studies, will have interesting applications in biological sciences.

### 2.8. Ion exchange resins

Polystyrene-based anion exchange resin microspheres had been successfully evaluated as a carrier system for the sustained delivery of the cytotoxic agent 5-FU. A batch ion exchange procedure was developed for drug vehiculization, showing that loading proceeded to near resin capacity [104].

## 3. Preclinical studies with 5-fluorouracil delivery systems

Up to know the development of 5-FU delivery systems had been mainly focussed on the study of the physicochemical properties and the *in vitro* behavior of these carriers. Very promising results are coming from *in vivo* studies: better biodistribution profiles (higher 5-FU accumulation at the target tissue, e.g., enhancement of 5-FU brain uptake) [92], significant improvement of the pharmacokinetic profile [15], and enhanced antitumor activity [13,14,15,28,29,30,34,35,36,37,40,41,43,61,62,63,64,65,66,67,68,69,72,73,74,92,99] in comparison to the free drug. These interesting results must encourage new efforts at the *in vitro* and *in vivo* stages of development of 5-FU delivery systems.

## 4. Conclusions

Concomitant research strategies for the development of specific 5-FU delivery systems to tumors has implemented a wide variety of approaches such as, parenteral, oral, topical and aerosolized formulations, vesicular systems, biodegradable micro and nanoparticles, or magnetic-responsive carriers, etc. Some of these strategies were also aimed at overcoming the rapid metabolization and drug resistance associated with 5-FU. Oral, topical and aerosolized formulations are proposed to treat local tumors, while other approaches are aimed at the systemic therapy of cancers. Micro and nanoparticulate systems had shown very promising results in the improvement of the pharmacokinetic profile of this antitumor drug and, simultaneously, in the enhancement of the anticancer action against experimental solid tumors.

Altogether, the delivery strategies adopted for 5-FU led to a considerable improvement in the treatment of cancers at the preclinical stage, and some of them are potential candidates for clinical trials (PLGA-based carriers, magnetic nanoparticles, etc.). However, more physicochemical and preclinical studies will allow the development of new surface-functionalized delivery systems, with higher 5-FU loading and sustained release capabilities, and able to improve the 5-FU uptake by tumor cells and the chemotherapeutic action of this antimetabolite drug.
